# Pediatric hypereosinophilic syndrome associated with liver damage, portal vein, splenic vein and superior mesenteric vein thromboses: a case report

**DOI:** 10.1186/s12887-023-04014-0

**Published:** 2023-05-12

**Authors:** Hai-Tao Zheng, Yan Xu, Xiao-Yu Yan, Yong-Bin Yan, Shu-Xia Ma, Ling-Ling Liu, Qian-Yi Zhao

**Affiliations:** 1 Department of Pediatrics, The First Affiliated Hospital of Henan University of Chinese Medicine, 19 Renmin Road, Zhengzhou, 450003 Henan China; 2 Henan University of Chinese Medicine School of Pediatrics, Zhengzhou, China; 3 Henan University of Chinese Medicine, Zhengzhou, China; 4 First Clinical Medical College of Henan University of Chinese Medicine, Zhengzhou, China

**Keywords:** Hypereosinophilic syndrome, Thrombocytopenia, Liver damage, Portal vein, Splenic vein, Superior mesenteric vein, Thrombosis

## Abstract

**Background:**

The hypereosinophilic syndrome (HES) is a group of rare blood disorders characterized by persistent eosinophilia and damage to multiple organs. HES can be either primary, secondary or idiopathic. Secondary HES are commonly caused by parasitic infections, allergic reactions or cancer. We described a pediatric case of HES associated with liver damage and multiple thrombi.

**Case summary:**

A 12-year-old boy with eosinophilia was complicated with severe thrombocytopenia, liver damage, portal vein, splenic vein, and superior mesenteric vein thromboses. The thrombi recanalized after treatment with methylprednisolone succinate and low molecular weight heparin. No side effects appeared after 1-month.

**Conclusions:**

Corticosteroids should be used at an early stage of HES to prevent further damage to vital organs. Anticoagulants should be recommended only in cases with thrombosis which should be actively screened as a part of evaluation of end organ damage.

The hypereosinophilic syndrome (HES) is a group of blood disorders characterized by an abnormal accumulation of eosinophils. Some varieties of HES have been described in families, while other types have been associated with infections, allergic diseases and cancer [1]. A recent study suggested that HES should be divided into different clinical subtypes depending on the presence of eosinophil abnormalities longer than 6 months and/or any damage in major organs [2]. Different HES subtypes might explain the heterogeneity of response to treatments.

This study reported a pediatric case of HES associated with liver damage and thromboses of the portal vein, the splenic vein and the superior mesenteric vein. Although we could not determine whether HES was caused by drugs or any infection before admission, we excluded allergies, tumors and parasitic infections. The thrombi were recanalized after administration of methylprednisolone succinate and low molecular weight heparin. All procedures were performed in compliance with institutional and/or national ethical standards and the Declaration of Helsinki (revised 2013).

## Case description

**Fig. 1:**
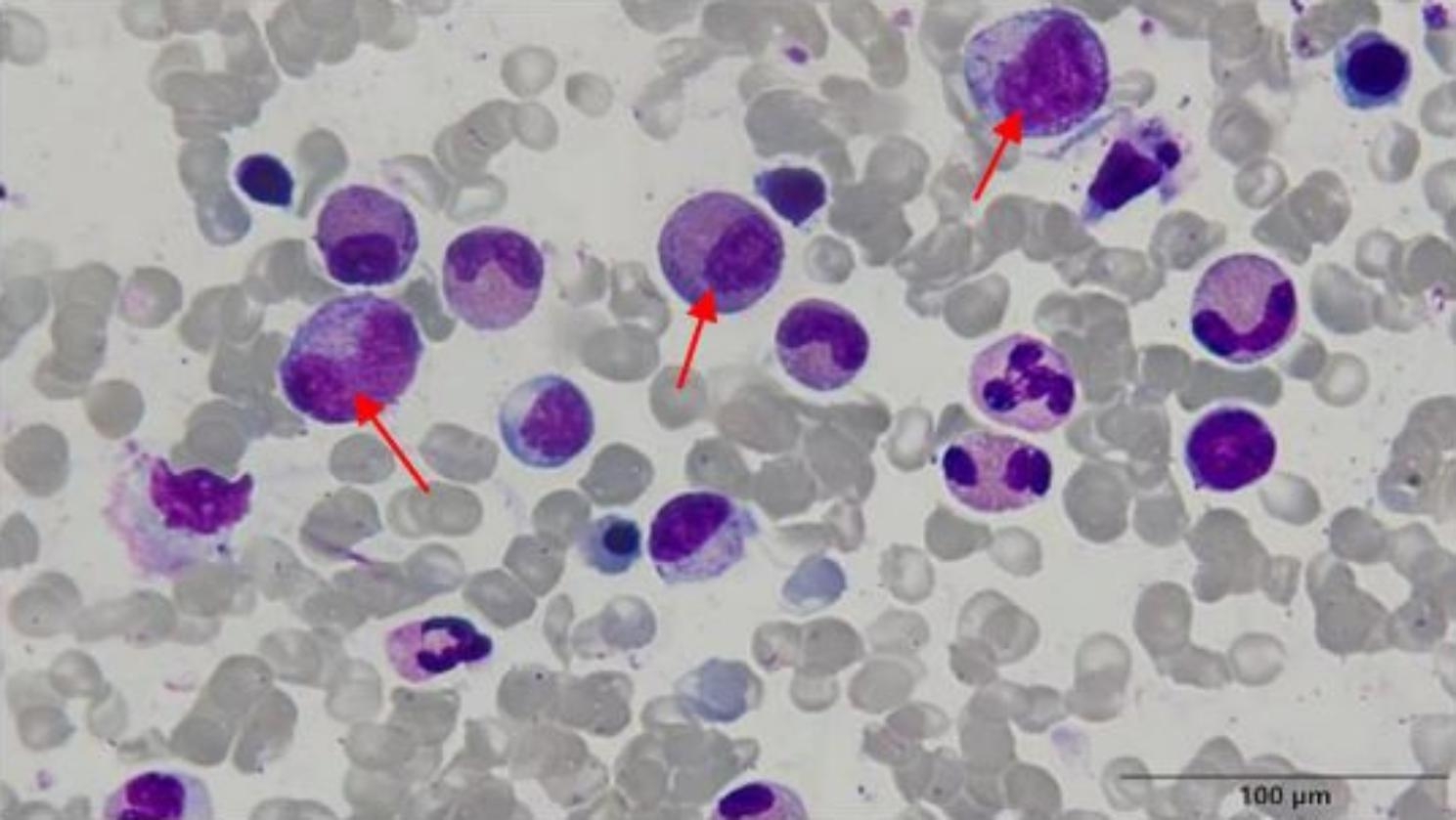
The bone marrow aspiration. The arrows point to eosinophils

A 12-year-old boy weighing 64 kg started complaining of headache associated with fever and abdominal pain. On the third day, the headache worsened and the parents took him to a local clinic. The child was treated with oral medications for an acute upper respiratory tract infection. During the following three days the headache aggravated severely. Nausea, vomiting and abdominal pain were also present. The body temperature was 37.2 °C. The local clinic stopped the oral medications and administered an intramuscular injection of an anti-inflammatory drug to the child. On the seventh day the symptoms did not improve, the body temperature rose to 38.2 °C. The boy was then transferred to a tertiary hospital. The blood examination was as follows: white blood cells: 17.82 × 10^9^/l (reference values: 4–10 × 10^9^/l), red blood cells: 5.34 × 10^12^/l (reference values: 3.5–5.5 × 10^12^/l), hemoglobin: 139 g/l (reference values: 109–146 g/l), platelets: 37 × 10^9^/l (reference values: 100–300 × 10^9^/l), eosinophil count: 5.22 × 10^9^/l (reference values: 0-0.45 × 10^9^/l), eosinophil percentage: 29.3% (reference values: 0.5-5), neutrophil percentage: 53.9% (reference values: 51–75), lymphocyte percentage: 11.7% (reference values: 20–40) and C-reactive protein: 17 mg/l (reference values: 0–5 mg/l). Liver function tests were as follows: alanine aminotransferase: 707 U/l (reference values: 0–40 U/l), aspartate aminotransferase: 398 U/l (reference values: 0–40 U/l) and the other tests were normal. The physical examination revealed scattered bleeding spots on the abdomen, the back and the dorsum of feet. The child was treated with injections of cefotaxime for 3 days without a specific diagnosis and with the patients prolonged complaints, together with proven thromboses. Methylprednisolone was intravenously infused for 1 day. After treatment, the body temperature was normal. The appetite improved, nausea and vomiting were relieved. The bleeding spots ameliorated. The next blood examination was as follows: white blood cells: 9.97 × 10^9^/l (reference values: 4–10 × 10^9^/l), red blood cells: 4.69 × 10^12^/l (reference values: 3.5–5.5 × 10^12^/l), hemoglobin: 119 g/l (reference values: 109–146 g/l), platelets: 53 × 10^9^/l (reference values: 100–300 × 10^9^/l), eosinophil count: 0.45 × 10^9^/l (reference values: 0-0.45 × 10^9^/l), eosinophil percentage: 0.45% (reference values: 0.5-5), neutrophil percentage: 72.8% (reference values: 51–75), lymphocyte percentage: 17.8% (reference values: 20–40) and C-reactive protein: 15 mg/l (reference values: 0–5 mg/l). The local hospital in view of the serious condition of the child and the complex, unknown cause, coupled with the guardian who was very worried about the condition of the child, referred the child to our hospital. On admission, he complained of abdominal pain with no nausea or vomiting. Scattered petechiae were present on the dorsum of his feet. No bruising, ecchymoses, epistaxis or gingival bleeding was observed. The family reported no previous history of allergies or exposure to drugs before the onset of symptoms. The child did not eat raw meat or unwashed fruits/vegetables before the appearance of headache. The personal and family health histories were not relevant. At the physical examination, no dry or wet rales were heard in the lungs. Heart sounds were normal. The abdomen was flat, no varicose veins were seen. The upper abdominal muscles were tense, with mild tenderness on palpation. No masses in the abdomen were noted, liver and spleen were not palpable under the ribs. The blood examination was as follows: white blood cells: 13.9 × 10^9^/l (reference values: 3.5–11.7 × 10^9^/l), red blood cells: 4.69 × 10^12^/l (reference values: 4.10–5.30 × 10^12^/l), hemoglobin: 131 g/l (reference values: 109–146 g/l), platelet count: 48 × 10^9^/l (manual method, reference values: 125–350 × 10^9^/l), neutrophil percentage: 33.8% (reference values: 40.0–75.0), lymphocyte percentage: 26.3% (reference values: 20.0–50.0), eosinophil percentage: 35.2% (reference values: 0.4-8.0), neutrophil count: 4.71 × 10^9^/l (reference values: 1.80–6.30 × 10^9^/l), lymphocyte count: 3.66 × 10^9^/l, (reference values: 1.10–3.20 × 10^9^/l) eosinophil count: 4.90 × 10^9^/l (reference values: 0.02–0.52 × 10^9^/l), mean platelet volume: 11.5 fl. (reference values: 6.5–12.0 fl.), platelet distribution width: 17.4 fl. (reference values: 15.0–17.0 fl.) and C-reactive protein: 18.9 mg/l (reference values: 0–4). The platelet morphology was normal. Alpha-fetoprotein, carcinoembryonic antigen, prostate-specific antigen, ferritin and growth hormone were not elevated in the blood. Immunoglobulins and complement C3 and C4 were normal. On flow cytometry, CD3^+^ T cells and CD3^+^CD8^+^ T cells were slightly increased (CD3^+^ T cells: 2430/µl, reference values: 1141–1880/µl; CD3^+^CD8^+^ T cells: 1091/µl, reference values: 393–742/µl). The D-dimer was 9.60 µg/ml (reference values: 0-0.3 µg/ml), fibrin degradation products were 88.61 µg/ml (reference values: 0–5 µg/ml). Immunoglobulin G, immunoglobulin A, immunoglobulin M, immunoglobulin E, complement C3, complement C4 were normal.The β-d-glucan test for fungus, the invasive fungal test and parasite inlcuding anti-Cysticercus cellulosae IgG antibody, anti-Paragonimus IgG antibody, anti-Sparganum IgG antibody, anti-Schistosoma japonicum IgG antibody, and anti-hydatid IgG antibody examinations were all negative. Systemic lupus erythematosus and hepatitis such as hepatitis B virus surface antigen, anti-hepatitis C virus antibody, anti-hepatitis E virus IgM antibody, and hepatitis A serologic test for assessing hepatitis B, C, E, and A infection were excluded. The results of bone marrow examination showing that the percentage of eosinophils increased, accounting for 35.5%. The bone marrow aspirate showed a moderate HES (Judgment standard: the absolute number of eosinophils was 1.5-5 × 10^9^ /l, accounting for 15–49% in the classification.) .The results of the bone marrow aspiration are shown in Fig. 1.

**Fig. 2:**
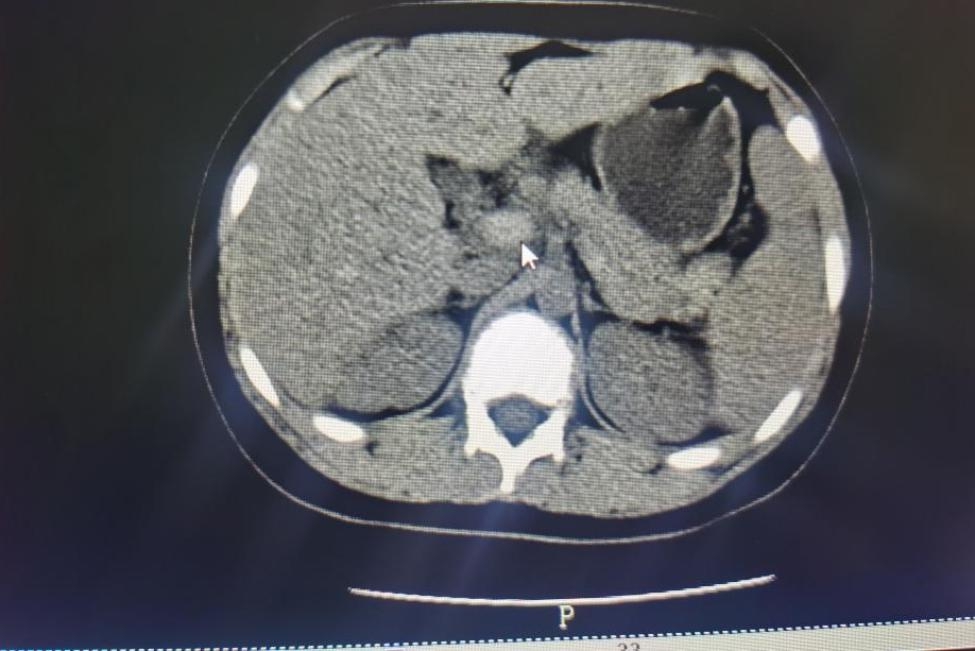
Arrows refer to tromboses in the main portal vein

**Fig. 3:**
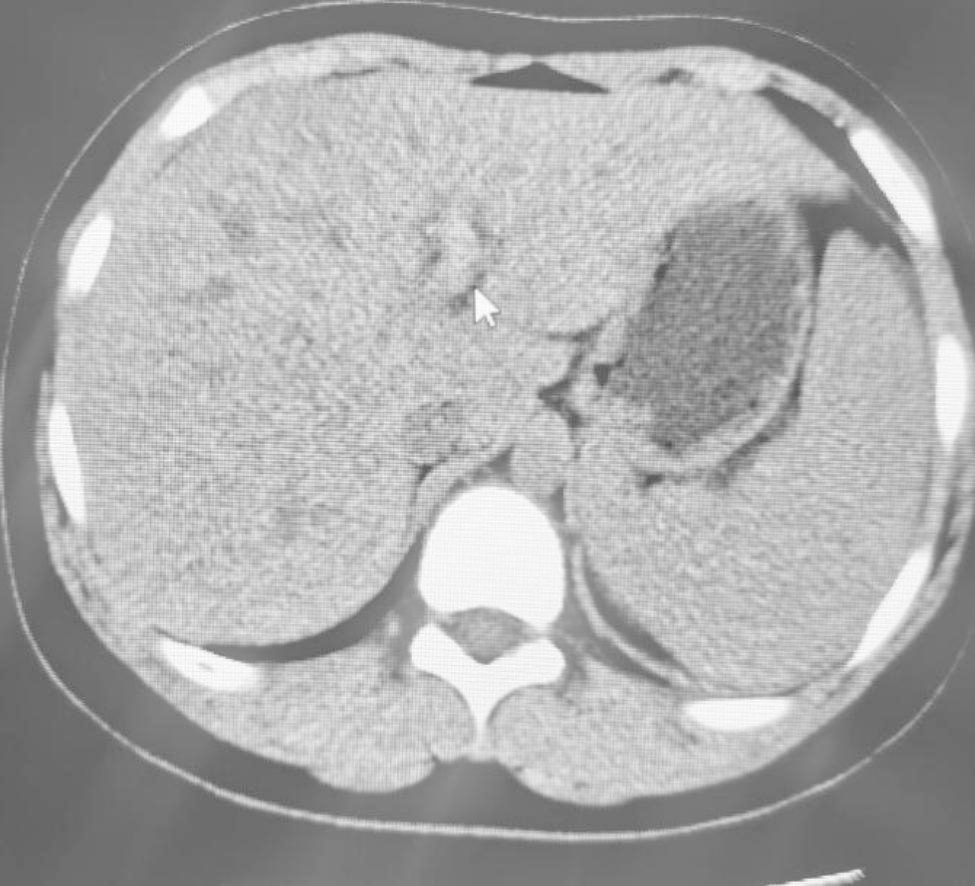
Arrows refer to tromboses in the left branch of portal vein

The abdominal computed tomography (CT) showed a thrombus and an increased lumen density of the trunk and branches of the portal vein, the splenic vein and the proximal superior mesenteric vein (Figs. 2 and 3). The spleen was enlarged, the gallbladder was inflamed with the presence of cholestasis. The CT of both lungs showed a few inflammatory foci in the right lung and an old nodule adjacent to the oblique fissure of the right lower lobe. The Doppler ultrasound showed no abnormalities in the gastrointestinal tract. A thrombosis of left and right branches of the portal vein was described, along with a thrombosis of the superior mesenteric vein and a thrombosis of the splenic vein. The liver was slightly enlarged with diffuse parenchymal echogenic changes. The volume of the gallbladder was increased. The wall of the gallbladder was thickened, while little biliary sludge was deposited inside the gallbladder. The magnetic resonance imaging of the brain showed an enlarged cisterna magna. The overall findings supported the diagnosis of HES complicated with thromboses of the portal vein, the splenic vein and the superior mesenteric vein. We administered injections of methylprednisolone succinate (60 mg daily), subcutaneous injections of low molecular weight heparin calcium (10,000 IU daily), ornidazole and rehydration therapy. The rash on the dorsum of his feet resolved completely after 6 days of treatment, and the platelet count rose to 128 × 10^9^/l. With the relief of abdominal pain symptoms, methylprednisolone succinate was reduced to 40 mg daily and then changed to 24 mg oral tablets. The treatment process is described in Table 1.

**Table 1:** Medications used during the hospitalization

Treatment	Admission Day	1	2	3	4	5	6	7	8	9	10	11	12	13	14	15	16	17	18	19	20	21	22	23	24	25
	Drugs																									
**Antibiotics**	Latamoxef Sodium for Injection	3000 mg daily																								
**Glucocorticoids**	Injections of methylprednisolone succinate					60 mg daily							40 mg daily													
	Oral methylprednisolone																			24 mg daily						
**Anticoagulant**	Low molecular weight heparin calcium			5000 IU daily		10,000 IU daily																				

**Fig. 4:**
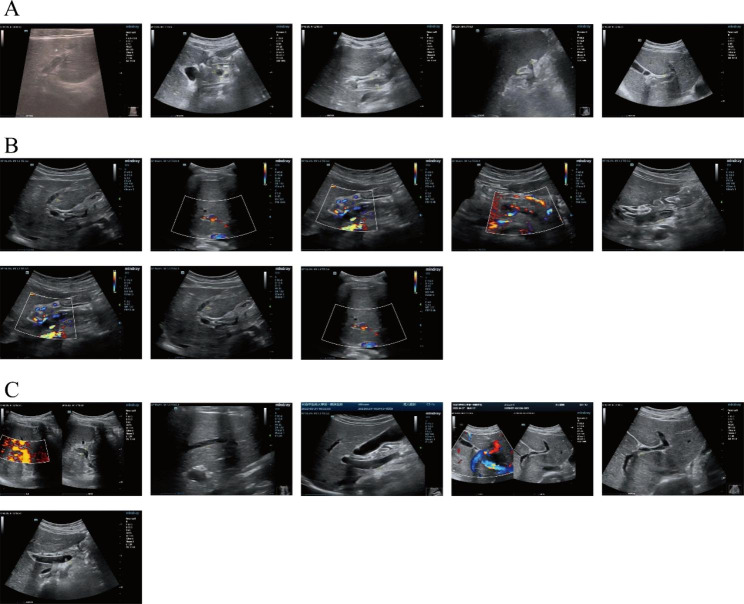
Thromboses of portal, splenic and superior mesenteric veins. **A** represents the Doppler ultrasound test on admission. **B** represents the Doppler ultrasound after 10 days. **C** represents the Doppler ultrasound test after 25 days

After 25 days, the abdominal pain disappeared. Liver function returned to normal. Signs of venous thromboses were still visible. During the following month, the child did not report any sequelae. The examination results are shown in Tables 2, 3 and 4; Fig. 4.

**Table 2:** Results of blood tests. WBC: white blood cells, RBC: red blood cells, PLT: platelets, NEUT: neutrophils, LYMPH: lymphocytes, EOS: eosinophils

Results	On admission	4th day	6th day	10th day	17th day	24th day	Reference range	Unit of measure
**WBC**	13.9	6.5	4.8	8.2	8.2	12.1	3.5–11.7	10^9^/l
**RBC**	4.69	4.57	4.39	4.74	4.60	4.90	4.10–5.30	10^12^/l
**PLT**	51	67	128	128	131	159	156–420	10^9^/l
**% NEUT**	33.8	23.0	43.5	43.6	40.2	72.9	40.0–75.0	%
**% LYMPH**	26.3	25.9	47.3	48.9	53.0	22.6	20.0–50.0	%
**% EOS**	35.2	40.2	0.9	3.0	1.1	0.2	0.4–0.8	%
**# EOS**	4.90	2.60	0.04	0.25	0.09	0.02	0.02–0.52	10^9^/l

**Table 3:** Results of liver enzymes tests. ALT: alanine aminotransferase, AST: aspartate aminotransferase

Results	On admission	1st day	4th day	8th day	18th day	25th day	Reference range	Unit of measure
**ALT**	707.0	464.0	218.6	68.1	110.7	67.4	0.0–50.0	U/l
**AST**	398.0	132.0	39.9	29.3	44.8	22,7	0.0–40.0	U/l

**Table 4:** Results of coagulation function tests. PT: prothrombin time, INR: international normalized ratio, D-D: D-Dimer, FDPs: fibrin degradation products

Results	On admission	1st day	4th day	8th day	Reference range	Unit of measure
**PT**	13.5	14.8	14.1	13.3	9.4–12.5	seconds
**INR**	1.24	1.36	1.30	1.23	0.08–1.20	
**D-D**	9.60	9.30	4.70	2.04	0.00-0.30	µg/ml
**FDPs**	88.61	78.31	24.67	12.90	0.00–5.00	µg/ml

## Discussion

HES is a rare heterogeneous disease manifesting as an unexplained persistent eosinophilia, with or without multiple tissue and organ damage. It can be divided into secondary (reactive), primary (clonal), and idiopathic HES. Infections and drug reactions are the most common secondary causes of HES [3]. However, local physician did not perform any blood test at the local clinic and the guardian did not remember any previous drug therapy. We cannot exclude the use of a drug as the cause of HES. The first blood test was performed at the tertiary hospital, revealing an increase in absolute eosinophil count. When the child was at our hospital, he showed no signs of hypersensitivity throughout the 25 days of treatment. We found no signs of fungal or parasitic infections. After an internal discussion with the medical team, we suspected the cause might be an unknown infection.

Previous studies reported that HES is often complicated by severe cardiac or cerebral thromboses [4]. The abdominal CT scan showed thrombi blocking the portal vein, the splenic vein and the superior mesenteric vein. A thrombosis with end-organ damage is rare in patients with HES. A single adult case of portal and superior mesenteric vein thrombosis was described in 2018 [5], while two other reports described a multiple organ infiltration associated with a deep vein thrombosis [6, 7]. We described a rare manifestation of HES in the childhood, reporting concomitant severe thrombocytopenia, liver damage and thromboses of the portal vein, splenic vein and superior mesenteric veins. The cause of thrombocytopenia was interpreted in the following manner: eosinophil increase resulted in extensive thrombosis, abnormal coagulation function (indicating hypercoagulability), and secondary fibrinolysis, leading to thrombocytopenia. Additional bone marrow examination showed no hematological system disorder. We recommend that corticosteroids should be started at an early stage in patients with signs and symptoms of HES, and combined with anticoagulants in cases of proven thrombosis may prevent further damage to vital organs.

Corticosteroids are the first-line therapy for most forms of HES. They can reduce abnormal levels of CD3^−^CD4^+^ cells often associated with eosinophilia. In addition, some studies recommended a maintenance dose of corticosteroids because they can effectively reduce HES-related complications [8]. Hydroxyurea is considered a second-line therapy due to its hematological and gastrointestinal toxicity [9]. Patients who do not respond to glucocorticoids or hydroxyurea may ultimately benefit from IFN-α [8, 9]. Imatinib, nilotinib, sorafenib, mepolizumab, erlizumab, alemtuzumab, cyclophosphamide and cyclosporine are other potential therapeutic drugs against HES [10]. We administered methylprednisolone succinate, achieving a satisfactory treatment effect. Glucocorticoids can inhibit the production of cytokines and chemokines, inducing apoptosis of eosinophils and alleviating eosinophil-mediated tissue and organ damages.

HES is a clinically diverse disease and can be idiopathic or associated with a variety of underlying diseases, including allergic, rheumatic, infective or neoplastic diseases. Furthermore, the etiology of eosinophilia can be primary (myeloid), secondary (lymphocyte-driven) or unknown. It is difficult to distinguish the HES subtype based on the clinical diagnosis. Currently, corticosteroids remain the first-line treatment for most subtypes, but the increasing availability of novel therapeutic agents, including tyrosine kinase inhibitors for distinct driver clonal aberrations and monoclonal antibodies only on clinical protocols for patients with life-threatening HES refractory to standard therapies or with eosinophilic granulomatosis with polyangiitis, has certainly changed the treatment approaches for HES [11]. Due to the low number of HES cases reported in children and the high heterogeneity among different individuals, it is necessary to individualize the treatment according to the etiology, disease progression, eosinophil level and organ damage.

## Conclusions

We described a pediatric case of HES complicated with liver damage and thromboses of the portal vein, the splenic vein and the superior mesenteric vein. The thrombi were recanalized and liver enzymes returned to normal after treatment with methylprednisolone succinate, low molecular weight heparin and antioxidants. This single case could facilitate early detection and intervention in patients with HES. Early recognition and management with steroids and anticoagulants is important to prevent serious complications like bowel ischemia. We recommend that anticoagulants, in cases of documented thrombosis, and corticosteroids should be used at an early stage.

## Data Availability

The datasets generated/analysed during the current study are available. The corresponding authors of Zhao Qianyi and Yan Yongbin are the Point of Contact for Data Availability Request.
